# Predicting Glioblastoma Cellular Motility from In Vivo MRI with a Radiomics Based Regression Model

**DOI:** 10.3390/cancers14030578

**Published:** 2022-01-24

**Authors:** Kellen Mulford, Mariah McMahon, Andrew M. Gardeck, Matthew A. Hunt, Clark C. Chen, David J. Odde, Christopher Wilke

**Affiliations:** 1 Department of Radiation Oncology, University of Minnesota, 516 Delaware St. SE, Minneapolis, MN 55455, USA; mulfo019@umn.edu; 2 Department of Biomedical Engineering, University of Minnesota, 312 Church St. SE, Minneapolis, MN 55455, USA; mcmah131@umn.edu (M.M.); garde046@umn.edu (A.M.G.); oddex002@umn.edu (D.J.O.); 3 Department of Neurosurgery, University of Minnesota, 420 Delaware St. SE, Minneapolis, MN 55455, USA; hunt0888@umn.edu (M.A.H.); ccchen@umn.edu (C.C.C.)

**Keywords:** glioblastoma, cellular motility, MRI, radiomics

## Abstract

**Simple Summary:**

A diagnosis of glioblastoma carries a uniformly dismal prognosis. Contributing to this is the near certain chance of aggressive tumor spread and recurrence following treatment. Tumor cell motility may provide one way to characterize the tendencies of glioblastomas to spread and recur. We sought to develop a non-invasive technique for assessing tumor cell motility using quantitative features derived from in vivo preoperative magnetic resonance imaging. Our regression model accurately predicted tumor cell motility in a cohort of participants with preoperative imaging who also had mean cellular motility calculated for their resected tumor cells from time-lapse videos. This work establishes the feasibility of non-invasively characterizing the kinetic properties of tumors and could be used to select patients for motility-targeting precision therapies.

**Abstract:**

Characterizing the motile properties of glioblastoma tumor cells could provide a useful way to predict the spread of tumors and to tailor the therapeutic approach. Radiomics has emerged as a diagnostic tool in the classification of tumor grade, stage, and prognosis. The purpose of this work is to examine the potential of radiomics to predict the motility of glioblastoma cells. Tissue specimens were obtained from 31 patients undergoing surgical resection of glioblastoma. Mean tumor cell motility was calculated from time-lapse videos of specimen cells. Manual segmentation was used to define the border of the enhancing tumor T1-weighted MR images, and 107 radiomics features were extracted from the normalized image volumes. Model parameter coefficients were estimated using the adaptive lasso technique validated with leave-one-out cross validation (LOOCV) and permutation tests. The R-squared value for the predictive model was 0.60 with *p*-values for each individual parameter estimate less than 0.0001. Permutation test models trained with scrambled motility failed to produce a model that out-performed the model trained on the true data. The results of this work suggest that it is possible for a quantitative MRI feature-based regression model to non-invasively predict the cellular motility of glioblastomas.

## 1. Introduction

Glioblastoma is the most common primary brain tumor in adults. These tumors are hallmarked by their aggressive behavior and near uniformly dismal prognosis, with a median overall survival of typically 1–2 years [1,2]. The clinical standard of care, which consists of surgical resection followed by concurrent radiotherapy and alkylating chemotherapy, has not substantially changed over the past 20 years [3,4]. One of the factors contributing to the poor prognosis of these tumors is the near-certain risk of intracranial recurrence within either the initial tumor bed or other intracranial sites [3,5].

The ability of glioblastoma cells to migrate and infiltrate healthy tissues has long been thought to contribute to the high rate of recurrence and metastasis [6]. Cell migration is a complex process, with numerous signaling pathways contributing to the process. As an example, Klank et al. revealed a biphasic relationship between CD44 expression on both tumor cell migration speed and overall prognosis, with the highest migration speeds in a mouse model correlating with the worst prognosis [7]. Naturally, cell migration has emerged as a target for new therapeutic strategies for glioblastoma [8,9,10]. Research on the ability of glioblastoma to grow and spread has, by necessity, occurred either in vitro or in animal models [11,12,13,14]. The motile characterization by these methods is time consuming and costly, which limits potential clinical applications. A non-invasive method to characterize the motile function of tumor cells would provide an early marker for patients who might benefit from therapies targeting the mechanical properties of glioblastoma.

Magnetic resonance imaging (MRI) provides a powerful non-invasive method for probing tissue structure and function. MRI scanners are ubiquitous in hospitals that treat glioblastoma, and an MRI scan is typically the first diagnostic imaging test performed on patients with a suspected brain tumor. More recently, high-throughput quantitative imaging features derived from the voxel values of an image have emerged as a useful technology for characterizing malignant tissues [15,16,17]. Radiomics studies have utilized shape, first-order statistical, and textural features to grade primary brain tumors, predict survival, and identify IDH-1 mutant tumors or tumor sub-regions [18,19,20,21].

The purpose of this study is to determine if the quantitative imaging features derived from the preoperative MR images of patients with glioblastoma could be used to form a predictive model of the cellular motility of the tumor. Such a model would allow for the non-invasive characterization of the mechanical properties of tumors, which could guide treatment decisions. To accomplish this, we collected tissue samples from resected glioblastomas for motility calculations and preoperative MRI scans for non-invasive characterization. Using these data, we developed a regression model that is able to predict the mean cellular motility of glioblastoma tumors, establishing a link between cellular-scale dynamics and tumor-scale shape features.

## 2. Materials and Methods

### 2.1. Patients

**Figure 1:**
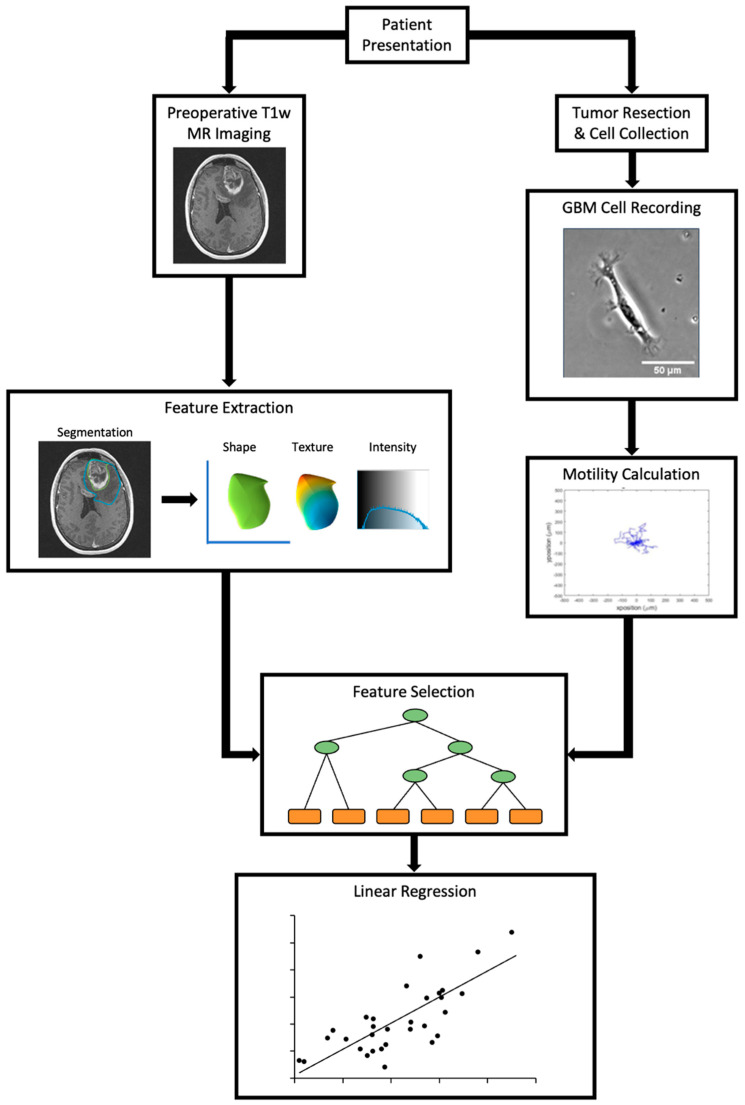
Flowchart detailing the methods described and used in the study. Patient cells isolated from resected tumor specimens were recorded and analyzed for motility values. Imaging features were derived from preoperative post-contrast T1w MR imaging.

A flowchart of the methods used in this study is shown in Figure 1. Patients treated for glioblastoma at the University of Minnesota Medical Center between the years 2017–2019 were identified for inclusion. The selection criteria included the presence of contrast-enhanced preoperative T1-weighted MRI scans and the availability of resected tissue for use in cell motility calculations. Patients were excluded if the resected specimen did not yield enough cells (N > 25 cells per condition) for motility characterization, T1-weighted post-contrast MR imaging was not available, or the MRI image quality was degraded due to the presence of an artifact. This study was approved by the University of Minnesota’s Institutional Review Board.

### 2.2. Live Cell Imaging and Motility Calculations

Briefly, tissue specimens from each patient were obtained during a surgical resection of the tumor and were submitted for routine surgical pathology analysis. Excess tissue for research purposes was then aseptically processed in the lab. The tissue sample was mechanically minced using a sterile scalpel and was transferred to a conical tube containing 15 mL 1× PBS. The specimen was then incubated in a water bath (37 °C) for 15 min, after which 10 mL of ACK lysing buffer (Gibco LSA1049201) was added, and the sample was incubated at room temperature for an additional 5 min. The sample was centrifuged at 220RCF for 5 min and was resuspended in phosphate-buffered saline (PBS). The sample was recentrifuged and resuspended in Neural Stem Cell (NSC) Media, which was composed of DMEM/F-12 (Gibco 11320033), 1× B-27 Supplement (Gibco 12587010), and 1× Pen/Strep (Corning 45000–650). The suspension was then filtered through a 40 µm cell strainer (CellTreat 229481) and was transferred to a Matrigel (Corning 354263) coated flask with 10 ng/ mL of EGF(Peprotech AF10015) and FGF(Peprotech AF10018B) and was incubated at 37 °C. Prior to imaging, the flasks were cultured until the majority of the debris and immune cells were removed by media exchanges (approximately 3–10 days). The cells were fed fresh media every 1–3 days, and the NSC media with the EGF and FGF growth factors were added every other day. All of the samples were processed within 16 h post-resection to maximize cell viability.

Motility estimates were calculated for each patient using the methodology previously detailed in Bangasser et al., 2017 [22]. Briefly, 12 mm polyacrylamide gels o that were ~50–100 µm thick and with Young’s modulus values of 0.7 kPa, 4.6 kPa, 9.3 kPa, 19.5 kPa, 100 kPa, and 200 kPa were cast onto glass-bottomed MatTek dishes (P12G-0-14-F, P35-0-20-C) and coated with laminin (163 µg/mL; Corning 354232). Approximately 10,000–15,000 cells were plated on each gel, and time-lapse phase-contrast movies were acquired by taking an image every 15 min for 16 h using a Nikon Ti2 or Nikon TiE Microscope with a 10×, 0.25 NA phase contrast objective lens. Cells were then tracked by an in-house segmentation and tracking algorithm. Motility was quantified by the random motility coefficient (RMC; units of µm^2^/min) and was calculated from the mean squared displacement of each cell over the imaging period [22]. The mean motility value for each tumor was obtained by averaging the individual cellular motility values across all of the gel stiffnesses that the cells were plated on.

### 2.3. Image Feature Extraction

All patients underwent pre-operative imaging with 3D T1-weighted post-contrast imaging sequences for surgical planning under a standard institutional protocol. The tumors were segmented on these scans, with the tumor volume consisting of the enhancing tumor without the inclusion of any surrounding edema. Additional sequences such as T2, FLAIR, and diffusion-weighted sequences were not included, as many of the patients had these studies performed at outside centers prior to referral to the University of Minnesota for treatment, and there was also a correspondingly large variance in the hardware, technique, and signal to noise ratio between centers. Contour accuracy was checked and verified by an experienced radiation oncologist specializing in the treatment of CNS tumors. The images were histogram-matched to the mean of the images to reduce mean signal-level variability. A total of 107 radiomics features were extracted from each tumor using the Pyradiomics software package [23]. Feature extraction histogram bin widths were set to produce 50–125 bins to increase the robustness of the extracted features. These imaging features included 32 shape and first order statistical features as well as higher order feature groups, including the grey-level co-occurrence matrix (N = 24), the grey-level run-length matrix (N = 16), the grey-level size zone matrix (N = 16), neighboring grey-tone difference matrix features (N = 5), and grey-level dependence matrix features (N = 14). A full list of the features included in the analysis can be found in the Pyradiomics documentation.

### 2.4. Feature Selection

Feature selection was complicated by the availability of many candidate variables to explain a smaller number of observations. In this study, feature selection was performed inside of the cross-validation scheme by ranking the contributions based on the response of each imaging feature in a bootstrap forest model, where 100 consecutive decision trees were trained on bootstrapped data samples [24]. Bootstrapping introduces a random component into the feature ranking process, so the bootstrap forest was repeated several times before feature selection could take place. The top 10-ranked features were selected for further variable selection using adaptive lasso regression.

### 2.5. Regression

Coefficient estimates for the selected variables were determined inside of the cross-validation scheme using adaptive lasso regression with cellular motility as the observed variable and with the quantitative imaging features serving as the explanatory variables [25]. While least squares regression produces a model with low bias but high variance, lasso regression introduces L1 regularization to the fitting procedure as a way of trading variance for bias [25]. Adaptive lasso modifies normal lasso regression by weighting the regularization parameter. In addition to having oracle properties, the adaptive lasso (similar to the lasso) can set parameter estimates to zero for variables that contribute little to the overall fit. In this way, the adaptive lasso acts as a second feature selection method. Limiting the number of variables in the model reduces the chance of overfitting on a limited number of instances.

### 2.6. Model Validation

Given the relatively small sample size of our dataset, leave-one-out cross-validation (LOOCV) was performed for model validation purposes. For each run, one subject was left out of the feature selection and fitting process. Altogether, the random forest feature selector and adaptive lasso regression were performed N times, where N = 31 is the total number of subjects. The root mean squared error across all the models trained in this way was calculated to approximate the generalization error of the model. In addition, the mean error determined from the adaptive lasso model was compared to a baseline model error, which is the error when the predicted motility was calculated to be the mean motility value of the training data. Finally, permutation tests, where the model training procedure is performed many times on scrambled motility data, were performed to assess whether the model was capturing noise rather than a relationship between imaging features and cellular motility.

## 3. Results

### 3.1. Demographics

In total, 31 patients (13 males/18 females) were included in this study. Twenty-five subjects (81%) were diagnosed with de novo glioblastoma, with the remaining six being diagnosed with recurrent disease. The median age at diagnosis was 57. An IDH-1 mutation was present in 2 out of the 31 patients (6%), while a total of 13 (42%) were found to have MGMT promoter methylation. The demographic information of the patients is summarized in Table 1.

### 3.2. Feature Selection

The ten features selected by the initial bootstrap forest are listed in Table 2. The adaptive lasso regression solution with the 10 variables selected using the bootstrap forest consistently eliminated six variables, leaving four for inclusion in the final model. The four features selected by the lasso regression procedure were (1) first-order 10th percentile, (2) first-order minimum, (3) gray-level run length matrix gray-level non uniformity, and (4) gray-level run length matrix long-run low gray-level emphasis. These four variables selected by the lasso were consistent throughout the LOOCV process. The *p*-values for the parameter estimates of each of these variables were <0.0001.

### 3.3. Regression

**Figure 2:**
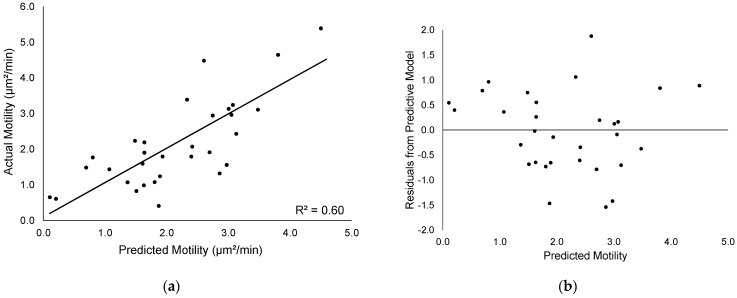
Model prediction of cancer cell motility. (**a**) A plot of the model-predicted mean motility values for each subject versus the actual motility. (**b**) Residual values between the predicted and actual motility values.

The average mean motility calculated from the time-lapse videos of the cells was 2.24 µm^2^/min, and the standard deviation was 1.25 µm^2^/min (N~192 cells per patient, 5945 cells total). To assess the performance of the image feature-derived model, the root mean square error (RMSE) of the imaging model was compared to that of a mean value predictor. The RMSE of the mean value predictor was 0.95 µm^2^/min. The image feature model produced with the adaptive lasso predicted the mean motility with an RMSE of 0.77 µm^2^/min. The R-squared value of the fitted line between the predicted motility values and the actual motility values was 0.60 (Figure 2a). The RMSE of the LOOCV validation process was 0.75 µm^2^/min. The residual values between the regression fit and the actual motility values exhibited no obvious correlation (Figure 2b).

### 3.4. Permutation Test

**Figure 3:**
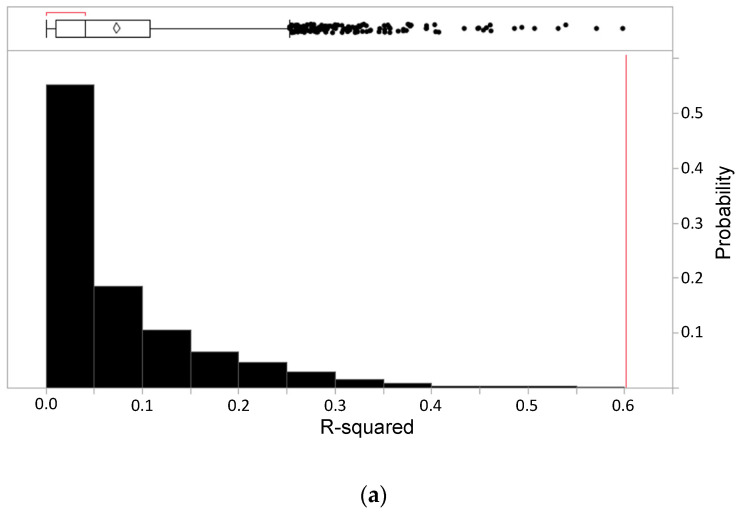

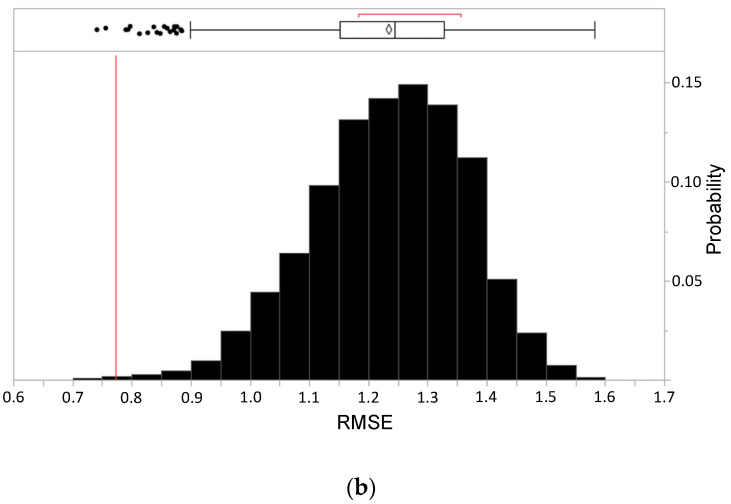
Testing of prediction significance using scrambled data. The results of permutation test training on the regression model were applied to scrambled cellular motility data. (**a**) The distribution of R-squared values from each model trained on a permutation of the motility data is lower than the observed R-squared in nearly all cases (*p* < 0.01). (**b**) The distribution of RMSE values from each model. The vertical lines indicate the performance of the original model, and nearly all RMSE values from the scrambled data are larger than the observed RMSE (*p* < 0.01).

To assess whether our model was capturing signal rather than noise, we performed a permutation test [26,27]. The imaging feature model was trained 2000 times on scrambled motility data. The distribution of the R-squared values and RMSE from the models constructed from the scrambled motility data are shown in Figure 3a,b, respectively. In comparison, the model obtained from the experimental results had a significantly higher R-squared value (*p* < 0.01) and lower RMSE (*p* < 0.01) compared to the scrambled data.

## 4. Discussion

This work represents an attempt to link quantitative MR imaging characteristics obtained in the clinic with mechanical cell properties measured by light microscopy in the lab. The model developed in this work predicts the mean cellular motility of glioblastoma tumors with an accuracy of 0.77 µm^2^/min. Given the maximum motility of ~5 µm^2^/min, the radiomic analysis enables the stratification of patient migration speeds into ~(5 µm^2^/min)/(0.77 µm^2^/min) = 6.5 levels of predicted of motility that are statistically independent of each other. The slightly smaller error from the LOOVC process points to the small sample size giving more weight to subjects that are near the edge of the selected parameter distributions. The regression model error is an improvement over the error of the baseline predictor, 0.95, which predicts all of the samples to be the mean of the training data. The lasso variable selection consistently selected the same four variables throughout the LOOCV process, suggesting that these features constitute a much stronger contribution to the model than the features selected by the random forest but not those selected by the lasso.

The sign of the parameter estimates for each of the four variables warrants discussion. The parameter estimate for the first-order 10th percentile feature is positive, suggesting that a tumor that presents a large fraction of hyperintense regions will have a higher motility. On MRI, this may present as a tumor with a wide enhancing ring. The coefficient estimate for the first-order minimum feature is negative, suggesting that a tumor with a more extreme hypointensity would have a higher motility value. A larger or more advanced tumor is more likely to present with necrosis or a cystic element, which would subsequently have a lower minimum voxel value. Tumor size at symptom onset is dependent on a variety of factors, including location and growth rate. Nonetheless, it may be reasonable to expect that a tumor that grows rapidly would have a higher motility value. We were unable to assess the growth rate on MRI in this particular patient cohort given the limited longitudinal imaging available; however, future work incorporating serial MRI data could potentially test this hypothesis. Overall, the high-rank variables all describe the range and variability of the MRI signal, with high range and variability correlating with faster cancer cell motility.

The grey-level non-uniformity feature calculated from the grey-level run-length matrix measures how dissimilar grey level runs are within the region of interest. A higher non-uniformity value practically translates to a higher degree of heterogeneity in the voxel values. The positive coefficient calculated for the non-uniformity feature in the regression model suggests that a tumor with a higher non-uniformity value has a higher mean motility value. Likewise, the coefficient for the long-run low gray-level emphasis is positive, indicating that a tumor that presents with more hypointensity runs (such as in a cystic or necrotic region) has a higher motility value.

This study has a few limitations. The first and most obvious is the small sample size of our dataset. Principally, a small sample size manifests itself as a decrease in model robustness, an increase in the variability of the feature selection process, and the tendency for individual outliers to have an outsized impact on the data. Additional data could be used to improve the generalizability of the model or would allow for prospective validation. The study sample used herein is roughly representative of national glioblastoma demographic statistics, with a median age of 57 and a higher proportion of males to females [28]. We only observed two cases of mutant IDH-1 out of 31, which is slightly lower than reported averages [29]. Even so, the model predictions are statistically significant as evidenced by the low R-squared and high RMSE after scrambling the data set. Further, many radiomics studies include features derived from additional MR contrasts. In this study, the MRI for surgical planning, which consisted of post-contrast 3D T1-weighted images, was the only sequence utilized.

The second limitation is that cell motility represents just one of several mechanical cellular characteristics that can characterize tumors. While previously published data suggest that cellular motility plays an important role in the growth and spread of tumors, a more comprehensive model taking proteomic or genomic factors into consideration may improve tumor characterization. For example, CD44 and glutamate levels are known to regulate migration speed through direct and indirect pathways, respectively [7,30]. Researchers developing therapeutics used to target these pathways specifically and may benefit more from a model that predicts the levels of these factors rather than the resultant migratory information. Additionally, motility on its own may not be predictive of prognosis or chance of recurrence, as cancer cell proliferation rates and the patient’s immune response need to be considered as well.

A third limitation is that glioblastoma tumors are known to be highly heterogeneous [31,32]. In this study, cellular motility was calculated from recordings of ~200 glioblastoma cells per patient derived from tumors containing 8 to 10 orders of magnitude more cells than that. Tumor cell motility may additionally vary from region to region within the tumor. Given the manner in which tissue procurement was performed, the specimens almost certainly contained a mixture of tumor subsites (i.e., core vs. leading edge). Similarly, the radiomics features were likewise derived from the entire tumor volume vs. tumor subsite. We are therefore unable to comment on the relative cellular motility/radiomic differences within tumors. Future work incorporating the biophysical properties of tumor subsites may help to further elucidate these relationships and, ultimately, may provide a way to predict where a tumor is likely to recur or where metastases may form.

Overall, this work represents one of the first efforts to connect the MR imaging-derived features of glioblastoma tumors to its dynamic single cell-level properties. Our model establishes a predictive correlation between several statistical features of the distribution of the voxel values of a tumor and the mean motility of the cells within that tumor. This study suggests that future therapeutics could potentially use imaging-based biomarkers to select patients who would benefit specific approaches to treating glioblastoma, especially those targeting cancer cell migration, the most.

## 5. Conclusions

In this study, quantitative image features from anatomical MRI datasets were used to develop a regression model that is able to non-invasively predict the cellular motility properties of glioblastoma tumors. Future work will seek to prospectively test the model developed here and will investigate the role of motility in patterns of recurrence, rates of tumor growth, and overall survival.

## Figures and Tables

**Table 1:** Patient characteristics.

Variable	Number of Patients
Sex (female/male)	13/18
Age–Median (Range)	57 (25–75)
De Novo GBM (Recurrent GBM)	25 (6)
IDH Mutation	2 (6.5%)
MGMT Promoter Methylation	13 (42.0%)
Location	
Frontal Lobe	13 (42.0%)
Temporal Lobe	11 (35.5%)
Parietal Lobe	5 (16.0%)
Occipital Lobe	2 (6.5%)
	Median (range)
Tumor Volume (cm^3^)	47.9 (0.8–118.7)

**Table 2:** Variable Selection.

Variable	Bootstrap Forest Rank	Adaptive Lasso Estimate	*p*-Value
First-Order 10th Percentile	1	1.671	<0.0001
First-Order Minimum	2	−4.230	<0.0001
GLRLM Gray Level Non-Uniformity	3	−0.00028	<0.0001
GLRLM Long-Run Low Gray-Level Emphasis	4	0.00395	<0.0001
GLSZM Grey-Level Variance	5	0	1.00
GLRLM Run Percentage	6	0	1.00
GLDM High Gray-Level Emphasis	7	0	1.00
GLSZM Zone Entropy	8	0	1.00
GLCM Joint Average	9	0	1.00
GLDM Dependance Non-Uniformity	10	0	1.00

## Data Availability

The radiomics features used to develop the model can be made available upon reasonable request and an approved data use agreement. Image data constitutes personal health information and will not be made available at this time.
